# Out-Patients and the War

**Published:** 1916-08-26

**Authors:** 


### OUT-PATIENTS AND THE WAR.

### The Experiences of Westminster Hospital.

It has been the experience of most of the large hospitals that ever since the beginning of the war the number of new out-patients has steadily declined. Similarly, when the total number of attendances for a given period during the war is compared with a corresponding period prior to the war, a decided decrease—although by no means so remarkable as in the case of new patients—is found to have taken place. This fact constitutes one of the noteworthy features of hospital work in time of war, and demonstrates how the war has relieved the pressure in one department while increasing it in others. A good example—no doubt a typical one—is furnished by the experiences of Westminster Hospital, the nature of which is clearly indicated by figures which the secretary has supplied to the writer.

### Some Remarkable Figures.

The following figures show the number of new out-patients who were treated at the hospital during the first three months of the last five years:—

| 1912 | 1913 | 1914 | 1915 | 1916 |
|---|---|---|---|---|
| 2,328 | 1,651 | 1,495 | 1,307 | 967 |

The figures for the first three months of 1912 and 1913 are included not because they can be used as a comparison with those for the corresponding period of 1915 and 1916 in showing the influence of the war on this department, but because, side by side with the other figures, they demonstrate in a striking way how the Insurance Act has relieved the hospital in an important branch of work. The pre-insurance figures are also useful as showing that while the effect of the war has been to reduce the number of out-patients in one quarter by approximately 500, the combined effect of the war and the Insurance Act has been to cause a quarterly diminution of over 1,300. To estimate the reduction that can be attributed solely to the influences of the war, the figures for 1915 and 1916 respectively must be compared with those for 1914, and then by comparing the statistics for the first three months of the present year with those for the first three months of last year, some idea may be obtained of the probable rate of the decrease as the war is prolonged.

### The Causes at Work.

It will be noticed that the difference between the numbers of out-patients in the first quarter of 1914 and 1915 is not nearly so marked as the difference between the figures for 1915 and 1916. The reason is not difficult to find. Although by the end of March last year the size of our Army had been vastly increased, the numbers of men drawn from the civil population did not begin to assume those large proportions to which we have now become accustomed until a later date, and it is doubtful whether even now the full effect of the exodus of the male population of military age has been felt by the hospitals. It is true that by the end of March this year the vast majority of the male population under forty-one years of age had joined the Colours, but the new Military Service Act will further deplete the ranks of possible hospital patients, and it is fairly safe to assume that the decline in the number of out-patients will continue until the last of the groups is called up.

It goes without saying that only to a limited extent is the diminished pressure in the out-patient department due intrinsically to the temporary absence from civil life of such a large proportion of the male population. Other related causes are in operation. For instance, the wage-earning power of the remaining males, individually and collectively, has increased; indeed, in some cases the wages earned are so enormously in excess of those obtainable in normal times that men who would formerly avail themselves of hospital treatment are now independent of both hospitals and panel doctors, and are prepared to pay for advice from medical practitioners and to buy their own medicines. Then, again, in a large proportion of cases the wives of men in the Forces are better off than when their husbands were in civil employment, and consequently they and their children are better fed, better clothed, and sometimes better housed; with less illness as a direct consequence. Then, again, the remarkable growth in the employment of women at more than a living wage has further depleted the number of possible hospital patients, while the diseases of the highly-paid munition workers are sometimes those of plenty rather than those of poverty, and as often as not can better be remedied by abstention from luxuries than by visits to the hospitals. Now, most of the factors which have contributed to the falling-off in out-patients are likely to operate so long as the war lasts, and consequently there is no reason to assume that the out-patient department will become normally active again until the war is over. But since we have now apparently reached the limit when transference from civil to military life becomes no longer necessary, and since the consequential factors are likely to remain normal, it would seem probable that the decline in the number of out-patients will from now onward become slower and slower until it finds a level where it will stop; and it may be assumed that this stage is not far away.

### Military Patients.

The same causes—or at least some of them—which have relieved the pressure on the out-patient department at Westminster Hospital no doubt account for the fact that although the number of beds available for ordinary in-patients in the cosy and home-like wards of this hospital has been reduced by eighty-six, to provide accommodation for sick and wounded soldiers, the waiting list is not heavier than usual, and apparently no hardship has been inflicted on the civil population in the district from which the hospital draws its patients.

